# The Social Gradient of Pain in Europe: Income‐Related Inequities and the Modifying Role of Osteoarthritis

**DOI:** 10.1002/ejp.70441

**Published:** 2026-11-01

**Authors:** Enrique Barrós‐Beracaza, Juan Antonio López‐Rodríguez

**Affiliations:** ^1^ Doctoral Programme in Epidemiology and Public Health Universidad Rey Juan Carlos Madrid Spain; ^2^ Member of the CiberAV Research Group Granada Spain; ^3^ Preventive Medicine and Public Health Department Rey Juan Carlos University Madrid Spain; ^4^ REDISSEC/RICAPPS Investigator‐Madrid Madrid Spain; ^5^ General Ricardos Primary Care Center‐Madrid Madrid Spain; ^6^ semFYC International Officer Barcelona Spain

**Keywords:** European health interview survey, health inequalities, income‐related inequality, osteoarthritis, pain prevalence, social determinants of health

## Abstract

**Background:**

Bodily pain is a frequent, disabling, and socially patterned population health burden. Although socioeconomic inequalities in pain have been described in Europe, it remains unclear whether this gradient changes in the presence of highly prevalent musculoskeletal conditions such as osteoarthritis.

**Methods:**

We conducted a cross‐sectional study using anonymized EHIS Wave 3 microdata from 2018 to 2020. The final sample included 278,250 adults from 30 European countries. Bodily pain in the past 4 weeks was analysed as a binary outcome. Household income quintile was the main exposure and self‐reported osteoarthritis the effect modifier. Adjusted prevalence ratios were estimated using robust Poisson regression with an income‐by‐osteoarthritis interaction. Adjusted probabilities, the Relative Index of Inequality, the Slope Index of Inequality, and between‐country heterogeneity were also estimated.

**Results:**

The weighted prevalence of pain was 55.04%. The income‐by‐osteoarthritis interaction was significant, *χ* ^2^(4) = 135.00; *p* < 0.001. Among adults without osteoarthritis, higher income was associated with lower pain prevalence (Q5 vs. Q1: aPR = 0.909; 95% CI: 0.893–0.925), whereas osteoarthritis was associated with substantially higher pain prevalence (aPR = 1.392; 95% CI: 1.370–1.413). Without osteoarthritis, the RII was 1.125 and the SII was 4.75 percentage points; with osteoarthritis, the RII was 0.964 and the SII was −2.21 percentage points. Between‐country heterogeneity was significant, *χ* ^2^(29) = 348.99; *p* < 0.001.

**Conclusions:**

Bodily pain affects more than half of European adults and follows an income‐related pattern. Osteoarthritis modifies this inequality: without osteoarthritis, pain follows a classic socioeconomic gradient, whereas with osteoarthritis, pain remains highly prevalent across income groups and the gradient is attenuated or slightly reversed.

**Significance Statement:**

This study demonstrates that income‐related inequalities in recent bodily pain vary according to self‐reported osteoarthritis status across 30 European countries. Its main contribution is to show that a high prevalence of pain in people reporting osteoarthritis may attenuate or obscure conventional socioeconomic gradients when pain is measured as a binary outcome. These findings highlight the limitations of relying exclusively on pain presence to characterize socioeconomic inequalities and support further investigation of pain severity and functional consequences.

## 1 Introduction

Pain is an unpleasant sensory and emotional experience associated with, or resembling that associated with, actual or potential tissue damage, and is shaped by biological, psychological, and social determinants (Raja et al. 2020). Pain represents a public health priority because of its frequency, functional and emotional impact, and socially unequal distribution (Todd et al. 2019; Zimmer et al. 2022, 2023). Epidemiologically, income should be considered both as an adjustment factor and as an axis for interpreting differences in risk exposure, coping resources, access to care, and health outcomes (Todd et al. 2019; Mackenbach et al. 2008; Zimmer et al. 2023). Among the clinical determinants of musculoskeletal pain in adults, osteoarthritis (OA) is especially relevant because of its association with persistent pain, impaired mobility, and disability (WHO 2023; Allen et al. 2022; Neogi 2013).

Population‐based evidence shows that pain is highly prevalent and socially patterned. The World Health Survey reported an age‐ and sex‐standardized pain prevalence of 27.5% across 52 countries, with substantial between‐country variation and higher likelihood among women and older adults (Zimmer et al. 2022). Similarly, the Gallup World Poll showed increasing self‐reported pain between 2009 and 2021, particularly among groups with lower educational attainment and fewer socioeconomic resources (Macchia 2022). Comparative evidence across 51 countries also found that lower wealth was consistently associated with higher pain probability, although this varied by macroeconomic context and income inequality (Zimmer et al. 2023).

In Europe, much of this burden presents as musculoskeletal pain, with persistent inequalities (Allen et al. 2022; Bimpong et al. 2022). The European Social Survey reported high 12‐month prevalences of back or neck, hand or arm, and foot or leg pain across 19 countries, with consistent socioeconomic inequalities. Bimpong et al. also observed higher pain frequency in women, reinforcing gendered and socioeconomic gradients (Bimpong et al. 2022). This gradient may be amplified when pain coexists with OA, a leading contributor to persistent pain, impaired mobility, disability, and reduced social participation (Steinmetz et al. 2023). Socioeconomic inequalities in pain extend beyond its prevalence and are also associated with greater pain intensity, functional interference, and pain‐related disability (Dorner et al. 2011; Lacey et al. 2013). Evidence further highlights the contribution of depression, obesity, and psychosocial factors to pain and associated disability (Bair et al. 2003; Chin et al. 2020; Solé et al. 2020).

Comparisons of pain prevalence across European countries are complicated by differences in outcome definitions, anatomical sites, severity thresholds, and reference periods. Population‐based studies have examined pain using different measures, including bodily pain in the preceding 4 weeks, chronic neck and low back pain, and chronic back pain (Ahrend et al. 2025; Huerta et al. 2025; Kuntz et al. 2017). These methodological differences limit the direct comparability of prevalence estimates. The European Health Interview Survey (EHIS) provides a harmonized framework for cross‐country analyses, including standardized measures of bodily pain in the past 4 weeks, household income, and self‐reported chronic conditions such as osteoarthritis (Eurostat 2020). Previous research using EHIS Wave 3 has demonstrated its application to the analysis of income‐related inequalities in cardiovascular risk factors across European countries (Pereira Silva et al. 2025). This framework also allows income‐related inequalities in recent bodily pain to be examined according to self‐reported osteoarthritis status.

Therefore, this study aimed to estimate the prevalence of pain in the past 4 weeks among adults aged 18 years or older in 31 European countries using EHIS Wave 3. It also analysed pain distribution across household income quintiles and examined whether OA modified the socioeconomic gradient in pain after accounting for sociodemographic, clinical, and contextual factors.

## 2 Methods

### 2.1 Study Design

We conducted an observational cross‐sectional study using anonymized microdata from the European Health Interview Survey (EHIS) Wave 3, collected between 2018 and 2020. The EHIS is a harmonised European population‐based survey designed to generate comparable cross‐country estimates of health status, chronic conditions, healthcare service use, social determinants of health, and sociodemographic characteristics.

The source population included adults living in private households across 31 European countries, including the European Union Member States participating in the survey, as well as Iceland, Norway, the United Kingdom, and Serbia. Data were collected using harmonized questionnaire‐based methods, including face‐to‐face interviews, telephone interviews, and self‐administered formats by postal mail or online, depending on the participating country (Eurostat 2020).

Detailed technical information on the variables, response categories, harmonization procedures, sampling design, and data collection methods is provided in the official Eurostat documentation for EHIS Wave 3 (Eurostat 2020). The manuscript was prepared in accordance with the STROBE recommendations for reporting cross‐sectional observational studies (Von Elm et al. 2008).

A study protocol and statistical analysis plan were developed before the statistical analyses were initiated, although neither document was prospectively registered in a publicly accessible repository.

### 2.2 Study Population

The study population comprised adults aged 18 years or older who were usual residents of private households in countries participating in the third wave of the European Health Interview Survey (EHIS). The initial dataset included 341,846 participants; after excluding individuals younger than 18 years and those without information on bodily pain in the past 4 weeks, 322,206 participants remained eligible. Participants with missing data on household income quintile, self‐reported osteoarthritis, covariates included in the final multivariable model, country of residence, or sampling weight were further excluded using a complete‐case approach. The final analytical sample included 278,250 participants from 30 European countries (Figure 1), with country‐specific sample sizes presented in Table S1.

**FIGURE 1:**
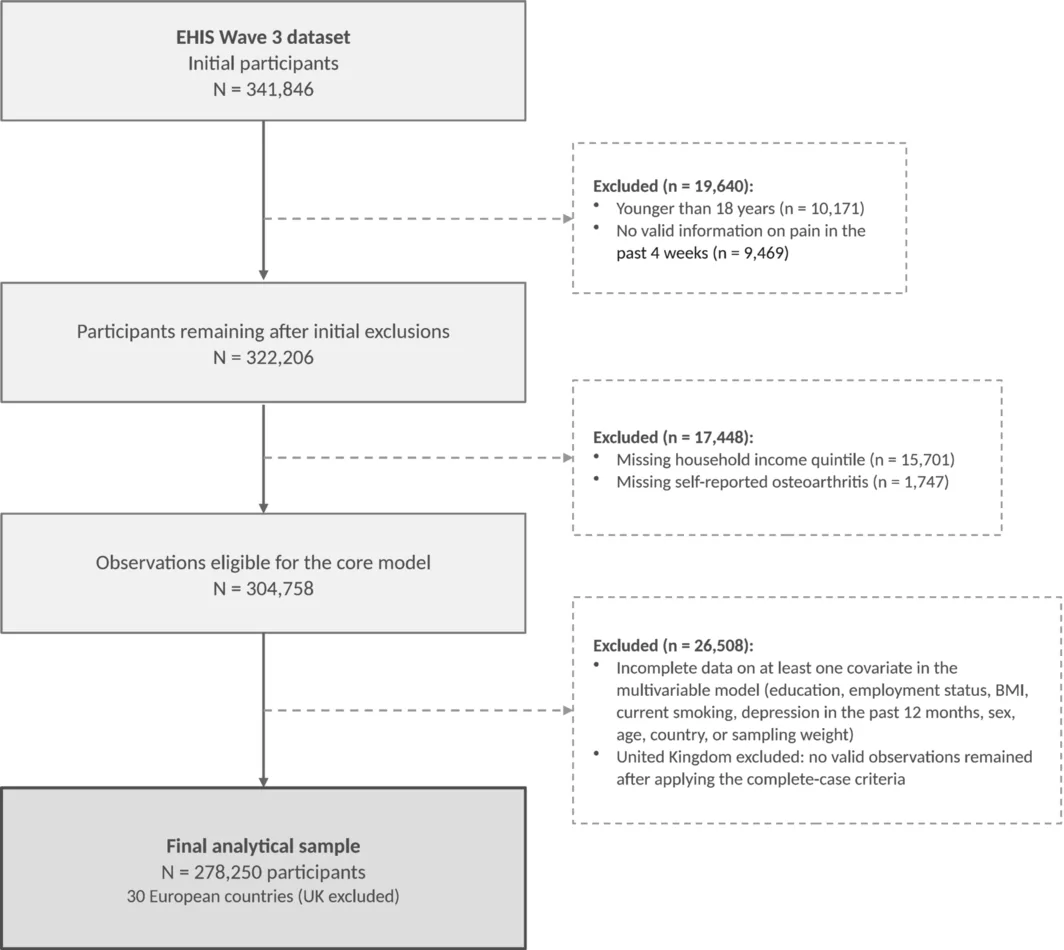
Flow diagram of participant selection (EHIS Wave 3). EHIS, European Health Interview Survey. Complete‐case criteria were applied to the covariates included in the final multivariable model. The United Kingdom was excluded from the final analytical sample because it did not contribute valid observations after applying these criteria.

### 2.3 Variables

#### 2.3.1 Outcome

The primary outcome was bodily pain in the past 4 weeks, assessed using the corresponding item from the European Health Interview Survey (EHIS) Wave 3. The variable was dichotomized as absence versus presence of pain. The response category ‘None’ was classified as absence of pain, whereas ‘Very mild,’ ‘Mild,’ ‘Moderate,’ ‘Severe,’ and ‘Very severe’ were grouped as presence of pain. This operationalization allowed us to estimate the population burden of recent bodily pain as a binary outcome, without distinguishing chronicity, anatomical location, specific clinical intensity, or functional interference (Eurostat 2020).

#### 2.3.2 Exposure and Effect Modifier

The main exposure was household income quintile, defined based on the net equivalized disposable income available in EHIS. This measure represents household income after taxes and social contributions, adjusted for household size and composition, and therefore provides an approximation of participants' relative economic position within each country. The variable was classified into country‐specific quintiles, from Q1, corresponding to the lowest income quintile, to Q5, corresponding to the highest income quintile. This operationalization allowed us to assess the socioeconomic gradient in pain in line with previous analyses of income‐related health inequalities in European populations (Eurostat 2020; Pereira Silva et al. 2025).

The primary effect modifier was self‐reported osteoarthritis, defined using the EHIS item assessing the presence of osteoarthritis during the past 12 months. The variable was recoded as a binary indicator of absence versus presence of osteoarthritis (Eurostat 2020).

#### 2.3.3 Covariates

Sex was classified as male or female. Age was grouped into five categories: 18–29, 30–44, 45–59, 60–74, and 75 years or older. Educational level was recoded into three categories according to the International Standard Classification of Education: low, corresponding to ISCED 0–2; medium, corresponding to ISCED 3–4; and high, corresponding to ISCED 5–8.

Employment status was harmonized into mutually exclusive categories: employed, unemployed, retired, unable to work, student, homemaker, and others. Body mass index was calculated from self‐reported height and weight and classified according to World Health Organization categories: underweight, normal weight, overweight, obesity Class I, obesity Class II and obesity Class III. Current smoking was recorded as current smoker versus non‐current smoker. Depression in the past 12 months was recorded as absence versus presence of self‐reported depression. (Eurostat 2020).

### 2.4 Statistical Analysis

The analytical sample was characterized according to the presence or absence of bodily pain in the past 4 weeks. Unweighted absolute frequencies and weighted percentages were reported for the sociodemographic, clinical, behavioural, and contextual variables included in the analysis. Bivariate comparisons between participants with and without pain were performed using weighted tests with robust variance estimation. Country‐specific distribution of the sample is presented in Table S1.

The sampling weight provided by EHIS was used after rescaling it to a mean of 1 to avoid extreme numerical magnitudes whereas preserving the relative relationships between weights. All analyses incorporated sampling weights and robust standard errors. Descriptive estimates were obtained using weighted procedures, and multivariable models were estimated using probability weights and robust variance.

To estimate the association between household income quintile and bodily pain, as well as the modification of this association by the presence of osteoarthritis, a Poisson regression model with robust variance was fitted. The model included quintile household income, self‐reported osteoarthritis, and the interaction term between these two variables. It was further adjusted for education, employment status, body mass index, current smoking, depression in the past 12 months, sex, age and country. Results were expressed as adjusted prevalence ratios (aPRs), with 95% confidence intervals (95% CIs) and two‐sided *p*‐values. The interaction between household income quintile and osteoarthritis was assessed using a joint test of the multiplicative interaction terms. (Zou 2004).

#### 2.4.1 Inequality and Geographical Heterogeneity

To quantify the socioeconomic gradient in pain beyond categorical comparisons across income quintiles, two summary measures of inequality were estimated: the Relative Index of Inequality (RII) and the Slope Index of Inequality (SII). These measures are commonly used in social epidemiology to assess relative and absolute health inequalities, respectively (Mackenbach and Kunst 1997; Moreno‐Betancur et al. 2015). For this purpose, an equidistant ridit score for household income quintile was constructed by assigning quintiles the values 0.1, 0.3, 0.5, 0.7 and 0.9, from the lowest to the highest income quintile. Because this coding directly estimates the contrast between higher and lower income, the RII was reported as its inverse to express the adjusted prevalence ratio between the lowest and highest ends of the income distribution.

The RII was estimated on the prevalence scale using Poisson regression with robust variance, including the ridit score for household income quintile, self‐reported osteoarthritis, and their interaction, with adjustment for education, employment status, body mass index, current smoking, depression in the past 12 months, sex, age and country. RII values > 1 indicate a higher adjusted prevalence of pain at the lowest income end of the distribution, whereas values below 1 indicate an inverse gradient.

The RII and SII estimates were reported stratified by osteoarthritis status. In addition, country‐specific models were fitted to estimate national RII and SII values, with the aim of describing the magnitude and direction of the socioeconomic gradient in pain on both relative and absolute scales. Estimates were transformed to the logarithmic scale, and their standard errors were derived from the corresponding 95% confidence intervals. For the SII, standard errors were estimated from the lower and upper limits of the 95% confidence interval.

#### 2.4.2 Sensitivity Analysis

As a sensitivity analysis, a logistic regression model was estimated using the same analytical sample and explanatory variables as the main multivariable model. The model included household income quintile, self‐reported osteoarthritis, and the interaction term between these two variables, with adjustment for educational level, employment status, body mass index, current smoking, depression in the past 12 months, sex, age and country.

Estimates from the sensitivity analysis were expressed as adjusted odds ratios (aORs), with 95% confidence intervals. Given the high prevalence of the outcome, the logistic regression results were interpreted exclusively as a complementary analysis to assess the directional consistency of the main findings, rather than as substitutes for the adjusted prevalence ratios estimated using the Poisson model with robust variance.

All analyses were conducted using Stata v19.5.

### 2.5 Ethical Considerations

EHIS was conducted in accordance with Regulation (EC) No. 1338/2008 of the European Parliament and of the Council, and Commission Regulation (EU) No. 141/2013 concerning statistics based on the European Health Interview Survey (EHIS). The survey was implemented by the competent national authorities in each participating country, in accordance with the applicable ethical, legal and statistical procedures. The present study used anonymized microdata provided by Eurostat for scientific research purposes, under the access, use, and confidentiality conditions established for this type of data. The dataset did not have direct personal identifiers. In addition, to reduce the risk of indirect identification, categories with fewer than 49 individuals were not reported.

## 3 Results

The EHIS Wave 3 dataset initially included 341,846 participants. After excluding individuals younger than 18 years of age (*n* = 10,171) and those without valid information on the main outcome variable, pain in the past 4 weeks (*n* = 9469), 322,206 participants remained. Participants with missing information on the main exposure, household income quintile (*n* = 15,701), and on the primary effect modifier, self‐reported osteoarthritis (*n* = 1747), were then excluded, leaving 304,758 observations eligible for the core model. Finally, after applying a complete‐case criterion for the covariates included in the multivariable model: education, employment status, body mass index, current smoking, depression in the past 12 months, sex, age, country and sampling weight, the final analytical sample included 278,250 participants from 30 European countries. The United Kingdom was excluded from the final analytical sample because it did not contribute valid observations after applying the complete‐case criteria required for the final multivariable model (Figure 1).

In the final analytical sample, the weighted prevalence of bodily pain in the past 4 weeks was 55.04%. Women represented 54.12% of the weighted population and were more commonly represented among participants with pain than among those without pain (58.88% vs. 48.29%; *p* < 0.001). Participants with pain were also older, with a higher proportion aged 75 years or older compared with those without pain (18.10% vs. 8.45%; *p* < 0.001) (Table 1).

**TABLE 1:** Characteristics of the study population according to pain in the past 4 weeks. Multivariable analytical sample, *n* = 278,250.

Variable	Category	Total, weighted %	No pain, (44.96) weighted %	Pain, (55.04) weighted %	*p*
Sex	Women	54.12	48.29	58.88	< 0.001
	Men	45.88	51.71	41.12	
Age group	18–29	11.82	16.44	8.04	< 0.001
	30–44	20.07	24.83	16.18	
	45–59	26.87	27.29	26.53	
	60–74	27.48	22.98	31.15	
	≥ 75	13.76	8.45	18.10	
Household income quintile	Q1, lowest	17.83	16.11	19.24	< 0.001
	Q2	20.02	18.26	21.46	
	Q3	20.72	20.51	20.89	
	Q4	20.96	22.18	19.96	
	Q5, highest	20.46	22.93	18.45	
Osteoarthritis	Without OA	82.53	94.35	72.88	< 0.001
	With OA	17.47	5.65	27.12	
Educational attainment	Low: ISCED 0–2	30.51	26.39	33.87	< 0.001
	Medium: ISCED 3–4	41.40	43.14	39.98	
	High: ISCED 5–8	28.09	30.46	26.15	
Employment status	Employed	48.47	55.71	42.56	< 0.001
	Unemployed	5.34	6.66	4.27	
	Retired	32.21	23.53	39.31	
	Unable to work	2.01	0.70	3.08	
	Student	4.23	5.89	2.88	
	Domestic tasks	5.43	5.47	5.40	
	Other	2.30	2.05	2.51	
Body mass index	Underweight	2.05	2.28	1.86	< 0.001
	Normal weight	42.99	48.22	38.71	
	Overweight	37.95	36.50	39.14	
	Obesity Class I	12.87	10.20	15.04	
	Obesity Class II	3.12	2.17	3.90	
	Obesity Class III	1.03	0.62	1.36	
Smoking status	Current smoker	22.50	24.37	20.97	< 0.001
	Non‐smoker	77.50	75.63	79.03	
Depression in the past 12 month	Depression	7.13	2.67	10.77	< 0.001
	No depression	92.87	97.33	89.23	

*Note:* Multivariable analytical sample: *n* = 278,250 participants from 30 European countries. Percentages are weighted estimates using wt_rescaled_m1. Unweighted outcome counts were 127,663 participants without pain and 150,587 participants with pain. *p*‐values correspond to design‐adjusted Pearson χ^2^ tests.

Self‐reported osteoarthritis was more frequent among participants who reported pain than among those who did not (27.12% vs. 5.65%; *p* < 0.001). Socioeconomic differences were also observed between the two groups: the lowest income quintile was more common among participants with pain than among those without pain (19.24% vs. 16.11%), whereas the highest income quintile was less frequent (18.45% vs. 22.93%; overall *p* < 0.001) (Table 1).

Participants with pain also had a higher proportion of low educational attainment (33.87% vs. 26.39%; overall *p* < 0.001), obesity Class I (15.04% vs. 10.20%), obesity Class II (3.90% vs. 2.17%), and obesity Class III (1.36% vs. 0.62%; overall *p* < 0.001 for BMI distribution), as well as depression in the past 12 months (10.77% vs. 2.67%; *p* < 0.001) (Table 1).

In the multivariable model, the interaction between household income quintile and self‐reported osteoarthritis was statistically significant (*χ* ^2^(4) = 135.00; *p* < 0.001), indicating that the association between income and pain differed according to osteoarthritis status. Among participants without osteoarthritis, an inverse socioeconomic gradient in pain was observed. Compared with the lowest income quintile (Q1), adjusted prevalence ratios progressively decreased as income increased: Q2 (aPR = 0.983; 95% CI: 0.968–0.999; *p* = 0.034), Q3 (aPR = 0.962; 95% CI: 0.946–0.977; *p* < 0.001), Q4 (aPR = 0.940; 95% CI: 0.925–0.956; *p* < 0.001), and Q5 (aPR = 0.909; 95% CI: 0.893–0.925; *p* < 0.001) (Figure 2).

**FIGURE 2:**
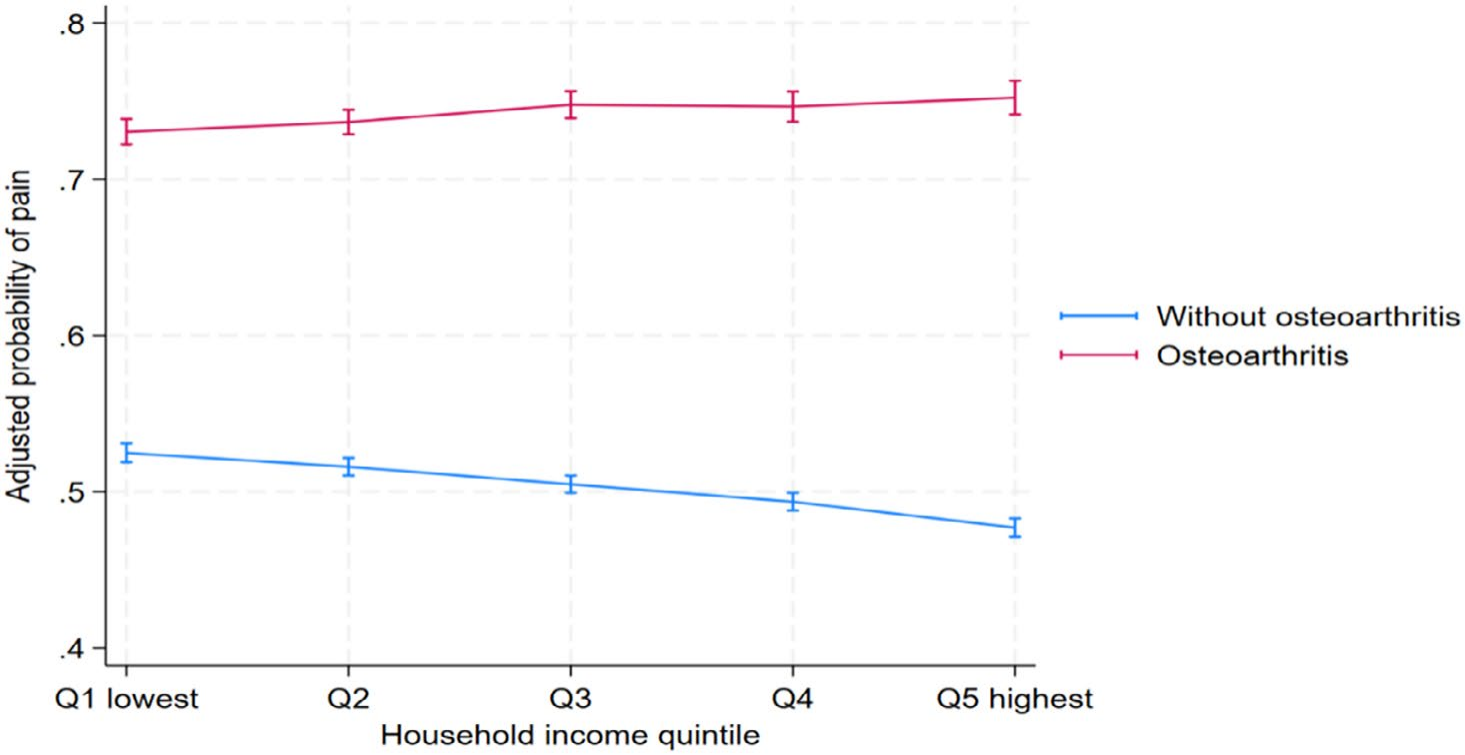
Adjusted probability of pain by household income quintile and osteoarthritis status. Estimates are adjusted predictive margins from the final multivariable model. Vertical bars represent 95% confidence intervals. Q1 and Q5 indicate the lowest and highest household income quintiles, respectively. The model was adjusted for sociodemographic, clinical, behavioural, and country‐level covariates.

Self‐reported osteoarthritis was associated with a higher adjusted prevalence of bodily pain (aPR = 1.392; 95% CI: 1.370–1.413; *p* < 0.001). The interaction terms between income and osteoarthritis were positive and statistically significant from Q2 through Q5, with increasing magnitudes from Q2 × osteoarthritis (aPR = 1.026; 95% CI: 1.005–1.047; *p* = 0.016) to Q5 × osteoarthritis (aPR = 1.133; 95% CI: 1.107–1.160; *p* < 0.001). In the adjusted model, higher pain prevalence was also observed among women (aPR = 1.146; 95% CI: 1.136–1.156; *p* < 0.001), adults aged 75 years or older (aPR = 1.479; 95% CI: 1.443–1.516; *p* < 0.001), individuals with depression in the past 12 months (aPR = 1.307; 95% CI: 1.294–1.320; *p* < 0.001), those with obesity Class III (aPR = 1.195; 95% CI: 1.162–1.229; *p* < 0.001), and participants unable to work due to long‐standing health problems (aPR = 1.308; 95% CI: 1.273–1.344; *p* < 0.001) (Table 2).

**TABLE 2:** Adjusted association between household income quintile, osteoarthritis, and pain in the past 4 weeks: Main model on the prevalence scale.

Variable	Category	aPR	95% CI	*p*
Household income quintile, stratum without osteoarthritis	Q1, lowest	1.000	Reference	—
	Q2	0.983	0.968–0.999	0.034
	Q3	0.962	0.946–0.977	< 0.001
	Q4	0.940	0.925–0.956	< 0.001
	Q5, highest	0.909	0.893–0.925	< 0.001
Osteoarthritis	Stratum with osteoarthritis versus without osteoarthritis	1.392	1.370–1.413	< 0.001
Interaction between household income quintile and osteoarthritis	Q2 × osteoarthritis	1.026	1.005–1.047	0.016
	Q3 × osteoarthritis	1.065	1.042–1.088	< 0.001
	Q4 × osteoarthritis	1.087	1.063–1.112	< 0.001
	Q5 × osteoarthritis	1.133	1.107–1.160	< 0.001
Educational attainment	Low: ISCED 0–2	1.052	1.041–1.063	< 0.001
	Medium: ISCED 3–4	1.000	Reference	—
	High: ISCED 5–8	0.975	0.964–0.986	< 0.001
Employment status	Unemployed	1.000	Reference	—
	Employed	1.055	1.031–1.079	< 0.001
	Retired	1.116	1.089–1.144	< 0.001
	Unable to work	1.308	1.273–1.344	< 0.001
	Student	1.006	0.969–1.045	0.738
	Domestic tasks	1.036	1.007–1.065	0.014
	Other	1.093	1.057–1.130	< 0.001
Body mass index	Normal weight	1.000	Reference	—
	Underweight	1.033	1.001–1.066	0.044
	Overweight	1.070	1.060–1.080	< 0.001
	Obesity Class I	1.134	1.121–1.148	< 0.001
	Obesity Class II	1.165	1.144–1.187	< 0.001
	Obesity Class III	1.195	1.162–1.229	< 0.001
Smoking status	Non‐smoker	1.000	Reference	—
	Current smoker	1.028	1.017–1.039	< 0.001
Depression in the past 12 months	No depression	1.000	Reference	—
	Depression in the past 12 months	1.307	1.294–1.320	< 0.001
Sex	Men	1.000	Reference	—
	Women	1.146	1.136–1.156	< 0.001
Age group	18–29	1.000	Reference	—
	30–44	1.148	1.122–1.173	< 0.001
	45–59	1.313	1.286–1.341	< 0.001
	60–74	1.351	1.320–1.382	< 0.001
	≥ 75	1.479	1.443–1.516	< 0.001

*Note:* Robust Poisson regression model weighted using wt_rescaled_m1. The model included country fixed effects and was adjusted for educational attainment, employment status, body mass index, current smoking, depression in the past 12 months, sex, and age. aPR: adjusted prevalence ratio. Analytical sample: *n* = 278,250. The joint test for interaction between household income quintile and osteoarthritis was statistically significant, χ^2^(4) = 135.00; *p* < 0.001.

Adjusted probabilities showed a similar pattern. Among participants without osteoarthritis, the adjusted probability of pain decreased from 0.525 in Q1 (95% CI: 0.519–0.531) to 0.477 in Q5 (95% CI: 0.471–0.483), corresponding to a difference of −4.78 percentage points (95% CI: −5.65 to −3.92; *p* < 0.001). Among participants with osteoarthritis, adjusted probabilities were higher and increased from 0.730 in Q1 (95% CI: 0.722–0.738) to 0.752 in Q5 (95% CI: 0.741–0.763), corresponding to a difference of +2.18 percentage points (95% CI: 0.86–3.51; *p* = 0.001) (Figure 2).

In the prevalence‐scale inequality model adjusted for the same covariates, the interaction between income ridit score and osteoarthritis was statistically significant (*χ* ^2^(1) = 137.61; *p* < 0.001), indicating that the socioeconomic gradient in pain differed by osteoarthritis status. Among participants without osteoarthritis, pain showed a positive socioeconomic gradient, with higher prevalence among lower‐income groups (RII = 1.125; 95% CI: 1.103–1.147; SII = 4.75 percentage points; 95% CI: 3.96–5.53). In contrast, among participants with osteoarthritis, the gradient was slightly inverse (RII = 0.964; 95% CI: 0.945–0.983; SII = −2.21 percentage points; 95% CI: −3.37 to −1.05) (Table 3).

**TABLE 3:** Socioeconomic inequality in pain by osteoarthritis status on the prevalence scale: Relative Index of Inequality and Slope Index of Inequality.

Stratum	RII	95% CI	*p*	SII, pp	95% CI	*p*
No osteoarthritis	1.125	1.103–1.147	< 0.001	4.75	3.96–5.53	< 0.001
Osteoarthritis	0.964	0.945–0.983	< 0.001	−2.21	−3.37 − 1.05	< 0.001

*Note:* RII represents the adjusted prevalence ratio and SII the adjusted absolute prevalence difference between the lowest‐ and highest‐income ends of the socioeconomic gradient. Models were adjusted for educational attainment, employment status, body mass index, current smoking, depression in the past 12 months, sex, age, and country fixed effects. Analytical sample: *n* = 278,250. The income‐ridit × osteoarthritis interaction was statistically significant, χ^2^(1) = 137.61; *p* < 0.001.

The socioeconomic gradient in pain also varied significantly across countries. The income × country interaction was statistically significant (*χ* ^2^(29) = 348.99; *p* < 0.001), as were the income × country and income × osteoarthritis × country interactions in the three‐way interaction model. In country‐specific analyses, 10 countries showed significant positive gradients on both the relative and absolute scales, with the largest relative gradients observed in Norway, Czechia, Slovakia, Romania and Austria. Serbia was the only country showing a significant inverse gradient on both scales. The remaining countries did not show significant gradients after multivariable adjustment.

These geographical differences are illustrated in the country‐specific RII maps for the overall analytical sample, participants without osteoarthritis, and participants with osteoarthritis, respectively (Figures S1a–S1c).

Meta‐analysis of national estimates confirmed substantial between‐country heterogeneity for both RII (*I* ^2^ = 59.38%; *p* = 0.0001) and SII (*I* ^2^ = 62.63%; *p* < 0.001). Overall, the meta‐analytic mean indicated a modest but significant positive socioeconomic gradient in pain, with a log‐RII of 0.053 (95% CI: 0.027–0.080; *p* = 0.0001) and an SII of 2.46 percentage points (95% CI: 1.23–3.70; *p* = 0.0001). Country‐specific RII estimates are displayed for the overall analytical sample and by osteoarthritis status in Figures S1a–S1c. Complete country‐specific and osteoarthritis‐stratified estimates are presented in Tables S3–S5.

The sensitivity analysis based on logistic regression, adjusted for the same covariates as the main model, showed that the observed associations remained in the same overall direction. The interaction between household income quintile and self‐reported osteoarthritis was statistically significant (*χ* ^2^(4) = 10.10; *p* = 0.0388). In the stratum without osteoarthritis, adjusted ORs were lower in the higher income quintiles, with the lowest value observed in Q5 (aOR = 0.840; 95% CI: 0.809–0.872; *p* < 0.001). Self‐reported osteoarthritis was associated with higher odds of bodily pain (aOR = 4.812; 95% CI: 4.420–5.240; *p* < 0.001). Complete estimates are presented in Table S2.

## 4 Discussion

This study shows that bodily pain in the past 4 weeks affects more than half of the European adults analysed and that its socioeconomic gradient is not uniform, but changes significantly according to the presence of self‐reported osteoarthritis. In a final analytical sample of 278,250 adults from 30 European countries, the weighted prevalence of pain in the past 4 weeks was 55.04%. Among adults without osteoarthritis, a classic socioeconomic gradient was observed, with a higher adjusted prevalence of pain in the lower income quintiles. By contrast, among those reporting osteoarthritis, the adjusted prevalence of pain was high across all income quintiles, and the gradient was attenuated or showed a slight reversal on both the relative and absolute scales. This supports the relevance of examining osteoarthritis as an effect modifier rather than only as a clinical covariate.

This prevalence may appear high when compared with estimates of chronic pain. This finding should be interpreted in light of the EHIS outcome definition, which includes all reported pain intensities, from very mild to very severe, without requiring a minimum duration beyond the four‐week reference period or evidence of functional interference. A comparable finding was reported in the German GEDA 2019/2020‐EHIS study, in which 58.8% of participants reported some degree of bodily pain during the preceding 4 weeks (Ahrend et al. 2025). Thus, the prevalence observed in our study is not inconsistent with other population‐based estimates using a similar measure, although differences in population characteristics and analytical methods preclude direct equivalence. Importantly, our estimate reflects recent bodily pain among participants included in the final analytical sample and should not be interpreted as the prevalence of chronic pain.

Population‐based studies have reported more pain among women, older adults, and lower socioeconomic groups, and a persistent relationship between social disadvantage, musculoskeletal pain, and poorer functional health (Bimpong et al. 2022; Eurostat 2020; Todd et al. 2019; Zimmer et al. 2022, 2023). In this study, pain was similarly concentrated among women, older adults, lower‐income and less‐educated participants, and those reporting osteoarthritis, obesity, or depression. Comparisons should be cautious because EHIS captures bodily pain during the past 4 weeks and does not identify chronic, disabling, or site‐specific musculoskeletal pain.

Osteoarthritis is a common cause of persistent pain, disability, and functional decline, with a growing population burden among adults and older people (Steinmetz et al. 2023; Van Schoor et al. 2016). Among participants without osteoarthritis, the RII was 1.125 indicating a higher adjusted prevalence of pain at the lowest income end of the distribution. Among those with osteoarthritis, the RII was 0.964, consistent with a slight reversal of both the relative and absolute gradients.

This reversal should not be interpreted as a clinical advantage for lower‐income groups, nor as evidence of an absence of inequality among people with osteoarthritis. Rather, it suggests that when the baseline prevalence of pain is very high, income may have reduced ability to discriminate the binary presence of pain. In this context, inequality may shift towards dimensions not captured by this outcome, including severity, functional interference, access to care, and quality of life (Fonseca‐Rodrigues et al. 2022; Neogi 2013; Yan et al. 2022).

Recent EHIS Wave 3 evidence on self‐medication among individuals with pain supports this interpretation, showing cross‐country variation and associations with pain severity, education, medicine availability and financial barriers. When pain burden is high, inequalities may be expressed less through symptom presence and more through management pathways, access to care, and therapeutic response (Yeamans et al. 2026).

The adjusted probabilities further support this interpretation. Among adults without osteoarthritis, the adjusted probability of pain decreased from 0.525 in the lowest income quintile to 0.477 in the highest income quintile. By contrast, among participants with osteoarthritis, adjusted probabilities were high across all income quintiles and remained within a narrow range, from 0.730 to 0.752. This pattern suggests that osteoarthritis shifts the pain burden towards high levels across the entire socioeconomic distribution, thereby flattening the classic gradient observed among participants without osteoarthritis. In this context, the presence of pain may become a less sensitive indicator for detecting inequalities within the osteoarthritis subgroup, where measures of severity, functional interference, disability, access to rehabilitation, and therapeutic response may be more informative.

These results extend the European evidence on pain and social inequality by integrating three dimensions that are often examined separately: socioeconomic position, musculoskeletal condition, and geographical variability. Previous studies have described social gradients in chronic low back pain, neck pain, musculoskeletal pain, and the overall pain burden across different European countries (Huerta et al. 2025; Kuntz et al. 2017; Maraschini et al. 2025). However, the present analysis contributes a harmonised multinational dataset, a common outcome definition, and an explicit assessment of osteoarthritis as a modifier of the socioeconomic gradient, rather than merely as a clinical covariate. This approach allows pain to be interpreted not only as an individual outcome, but also as a clinical‐social manifestation whose distribution depends on the interplay between social vulnerability and musculoskeletal burden.

Another relevant contribution is the formal demonstration of geographical heterogeneity in the socioeconomic gradient in pain. The interaction between income and country was statistically significant, and the three‐way interaction between income, osteoarthritis, and country showed that the osteoarthritis‐related modification of the gradient also differed across national contexts. In addition, meta‐analyses of the national estimates showed heterogeneity for both the RII and the SII. These findings indicate that Europe should not be interpreted as a homogeneous block in the social epidemiology of pain. The gradient may depend on national differences in social structure, working conditions, population ageing, osteoarthritis diagnosis, musculoskeletal care, rehabilitation, pain treatment and symptom reporting. However, these differences should not be causally attributed to health or social protection systems without macrostructural variables. This caution is consistent with the literature on health inequalities in Europe, which has shown the importance of social determinants and national contexts in shaping health gradients (Mackenbach et al. 2008; Pereira Silva et al. 2025).

Methodologically, a prevalence‐scale model was appropriate for a frequent outcome. In cross‐sectional studies with highly prevalent outcomes, odds ratios may overestimate the magnitude of associations and are often less intuitive than prevalence ratios (Barros and Hirakata 2003; Pearce 2004; Thompson et al. 1998). The sensitivity analyses based on logistic regression were consistent with the overall direction of the primary model, although estimates on the odds scale were larger in magnitude. Therefore, interpretation should rely mainly on the robust Poisson model, adjusted probabilities, and prevalence‐scale inequality indices. The combined use of the RII and SII allowed inequality to be described in both relative and absolute terms, an approach recommended in studies of social inequalities in health (Mackenbach and Kunst 1997; Regidor 2004).

### 4.1 Strengths and Limitations

A key strength is the integration of social inequality, osteoarthritis and geographical heterogeneity within one epidemiological framework. Using a harmonized European population survey allowed analysis of adults from 30 countries with comparable pain and social determinant measures. Osteoarthritis was examined as an effect modifier rather than only as an adjustment covariate, allowing assessment of whether income‐related inequalities differed in the presence of a clinically relevant musculoskeletal condition. The combined use of adjusted prevalence ratios, marginal probabilities, RII, SII, interaction tests, country‐specific estimates, and meta‐analytic summaries provided a comprehensive characterization of the gradient.

This study has limitations. Its cross‐sectional design precludes establishing temporality between income, osteoarthritis, and pain; therefore, it cannot determine whether socioeconomic disadvantage precedes pain, pain contributes to socioeconomic decline, or both relationships coexist. In addition, both pain and osteoarthritis were self‐reported, which may introduce misclassification and differences in interpretation across countries, languages, and cultures. The primary outcome grouped any bodily pain in the past 4 weeks, ranging from mild to severe pain. This increases the population sensitivity of the indicator but limits its clinical specificity and does not allow acute, recurrent, and chronic pain to be distinguished. Similarly, self‐reported osteoarthritis does not capture anatomical site, radiographic severity, duration, treatment, or functional impact, and may be influenced by diagnostic access, health literacy and clinical recognition.

Although the study protocol and statistical analysis plan were prepared before the analyses, they were not publicly preregistered. This limits the possibility of independently verifying the timing and content of the prespecified analytical decisions.

Other limitations relate to the measurement of socioeconomic position and the structure of the data. Income was operationalized as relative quintiles within each country, which allows within‐country gradients to be examined but does not capture absolute differences in living standards between countries. The complete‐case analysis may have introduced bias if missingness was not completely random, particularly for clinical and behavioural variables. The United Kingdom did not contribute valid complete observations after applying the criteria required for the final multivariable model and was not included in the final analytical sample. In addition, although the rescaled sampling weight was incorporated, the analytical dataset did not include explicit stratification variables or primary sampling units; consequently, it was not possible to fully reproduce the original complex sampling design of each national survey.

The interpretation of geographical heterogeneity also requires caution. Although the interaction between income and country, and the three‐way interaction between income, osteoarthritis and country, were formally evaluated, the study did not incorporate macrostructural variables that could explain why gradients differ across national contexts. Therefore, country‐specific analyses characterize geographical variability in the gradient, rather than causally explaining its determinants. Moreover, analyses stratified by osteoarthritis within each country were exploratory and showed lower precision in subgroups, especially where the number of participants with osteoarthritis was small.

### 4.2 Future Research Directions

Future research should move beyond cross‐sectional associations and examine longitudinal trajectories linking socioeconomic disadvantage, osteoarthritis onset and progression, pain persistence, and functional decline. Such studies should characterize pain not only as a binary outcome, but as a multidimensional clinical experience, including intensity, duration, anatomical distribution, interference with daily activities, disability, healthcare use, and treatment response. Comparative studies should incorporate country‐level determinants, including healthcare coverage, social protection, labour‐market conditions, income inequality, rehabilitation access, and specialised pain services. This would help clarify how individual vulnerability and structural context jointly shape pain burden and identify clinically and policy‐relevant targets for reducing inequalities in musculoskeletal pain.

## 5 Conclusion

This study shows that bodily pain in the past 4 weeks represents a substantial population‐level burden among European adults, with a distribution shaped by both socioeconomic position and self‐reported osteoarthritis. Osteoarthritis was associated with a higher prevalence of pain and modified the income‐related gradient: among adults without osteoarthritis, pain followed a conventional socioeconomic pattern, with higher prevalence in lower‐income groups, whereas among those with osteoarthritis this gradient was attenuated and slightly reversed.

The observed geographical heterogeneity indicates that inequalities in pain are not explained by individual‐level factors alone and should not be interpreted as a uniform European pattern. These findings support the integration of bodily pain into the epidemiological surveillance of health inequalities, incorporating socioeconomic position, musculoskeletal conditions, and national context. From a clinical and public health perspective, reducing the burden of pain requires not only identifying and assessing pain and related functional limitations but also addressing unequal access to timely, effective, and person‐centred care for musculoskeletal conditions.

## Author Contributions

Conceptualization: Barrós‐Beracaza, Enrique; López‐Rodríguez, Juan Antonio. Software: Barrós‐Beracaza, Enrique. Validation: López‐Rodríguez, Juan Antonio. Formal analysis: Barrós‐Beracaza, Enrique; López‐Rodríguez, Juan Antonio. Data curation: Barrós‐Beracaza, Enrique; López‐Rodríguez, Juan Antonio. Writing – original draft preparation: Barrós‐Beracaza, Enrique. Writing – review and editing: Barrós‐Beracaza, Enrique; López‐Rodríguez, Juan Antonio. Visualization: López‐Rodríguez, Juan Antonio. Supervision: López‐Rodríguez, Juan Antonio. Project administration: Barrós‐Beracaza, Enrique; López‐Rodríguez, Juan Antonio. All authors have read and agreed to the published version of the manuscript: Barrós‐Beracaza, Enrique; López‐Rodríguez, Juan Antonio.

## Funding

The authors have nothing to report.

## Conflicts of Interest

The authors declare no conflicts of interest.

## Supporting information


**Table S1:** Distribution of the analytical sample by country and presence of pain in the past 4 weeks.
**Table S2:** Sensitivity analysis using logistic regression of the association between household income quintile, osteoarthritis, and pain in the past 4 weeks.
**Table S3:** Relative inequality in pain by household income quintile and country: adjusted RII from the final multivariable model.
**Table S4:** Absolute inequality in pain by household income quintile and country: adjusted SII from the final multivariable model.
**Table S5:** Relative inequality in pain by household income quintile, country, and osteoarthritis status: adjusted RII from the final multivariable model.
**Figure S1a:** Relative inequality in bodily pain among participants with osteoarthritis.
**Figure S1b:** Relative inequality in bodily pain across European countries.
**Figure S1c:** Relative inequality in bodily pain among participants without osteoarthritis.
**Figure S2:** Forest plot of the Relative Index of Inequality for pain by country.
**Figure S3:** Forest plot of the Slope Index of Inequality for pain by country.
**Figure S4:** Forest plot of the Relative Index of Inequality for pain by country, stratified by osteoarthritis status.

## References

[ejp70441-bib-0001] Ahrend, J. N. , K. Jobski , C. Bantel , and F. Hoffmann . 2025. “Pain Intensity and Comorbid Depressive Symptoms in the General Population: An Analysis of the German Health Update Study (GEDA 2019/2020‐EHIS).” European Journal of Pain (London, England) 29: e4745. 10.1002/ejp.4745. PMC11755701 39444226

[ejp70441-bib-0002] Allen, K. D. , L. M. Thoma , and Y. M. Golightly . 2022. “Epidemiology of Osteoarthritis.” Osteoarthritis and Cartilage 30: 184–195. 10.1016/j.joca.2021.04.020. PMC10735233 34534661

[ejp70441-bib-0003] Bair, M. J. , R. L. Robinson , W. Katon , and K. Kroenke . 2003. “Depression and Pain Comorbidity: A Literature Review.” Archives of Internal Medicine 163: 2433–2445. 10.1001/archinte.163.20.2433 14609780

[ejp70441-bib-0004] Barros, A. J. , and V. N. Hirakata . 2003. “Alternatives for Logistic Regression in Cross‐Sectional Studies: An Empirical Comparison of Models That Directly Estimate the Prevalence Ratio.” BMC Medical Research Methodology 3: 21. 10.1186/1471-2288-3-21. PMC521200 14567763

[ejp70441-bib-0005] Bimpong, K. , K. Thomson , C. L. Mcnamara , et al. 2022. “The Gender Pain Gap: Gender Inequalities in Pain Across 19 European Countries.” Scandinavian Journal of Public Health 50: 287–294. 10.1177/1403494820987466. PMC8873965 33568013

[ejp70441-bib-0006] Chin, S.‐H. , W.‐L. Huang , S. Akter , and M. Binks . 2020. “Obesity and Pain: A Systematic Review.” International Journal of Obesity 44: 969–979. 10.1038/s41366-019-0505-y. 31848456

[ejp70441-bib-0007] Dorner, T. E. , J. Muckenhuber , W. J. Stronegger , É. Ràsky , B. Gustorff , and W. Freidl . 2011. “The Impact of Socio‐Economic Status on Pain and the Perception of Disability due to Pain.” European Journal of Pain 15: 103–109. 10.1016/j.ejpain.2010.05.013. 20558096

[ejp70441-bib-0008] Eurostat . 2020. European Health Interview Survey (EHIS Wave 3) — Methodological Manual (Re‐Edition 2020). Publications Office of the European Union.

[ejp70441-bib-0009] Fonseca‐Rodrigues, D. , A. Rodrigues , T. Martins , et al. 2022. “Correlation Between Pain Severity and Levels of Anxiety and Depression in Osteoarthritis Patients: A Systematic Review and Meta‐Analysis.” Rheumatology 61: 53–75. 10.1093/rheumatology/keab512. 34152386

[ejp70441-bib-0010] Huerta, M. Á. , A. Salazar , and J. A. Moral‐Munoz . 2025. “Trends in Chronic Neck and Low Back Pain Prevalence in Spain (2006–2020): Differences by Sex, Age, and Social Class.” European Spine Journal 34: 1331–1340. 10.1007/s00586-025-08676-5. 39928135

[ejp70441-bib-0011] Kuntz, B. , J. Hoebel , J. Fuchs , H. Neuhauser , and T. Lampert . 2017. “Social Inequalities in the Prevalence of Chronic Back Pain Among Adults in Germany.” Bundesgesundheitsblatt, Gesundheitsforschung, Gesundheitsschutz 60: 783–791. 10.1007/s00103-017-2568-z. 28516263

[ejp70441-bib-0012] Lacey, R. J. , J. Belcher , and P. R. Croft . 2013. “Does Life Course Socio‐Economic Position Influence Chronic Disabling Pain in Older Adults? A General Population Study.” European Journal of Public Health 23: 534–540. 10.1093/eurpub/cks056. PMC3719471 22874735

[ejp70441-bib-0013] Macchia, L. 2022. “Pain Trends and Pain Growth Disparities, 2009–2021.” Economics and Human Biology 47: 101200. 10.1016/j.ehb.2022.101200. 36423490

[ejp70441-bib-0014] Mackenbach, J. P. , and A. E. Kunst . 1997. “Measuring the Magnitude of Socio‐Economic Inequalities in Health: An Overview of Available Measures Illustrated With Two Examples From Europe.” Social Science & Medicine 44: 757–771. 10.1016/S0277-9536(96)00073-1. 9080560

[ejp70441-bib-0015] Mackenbach, J. P. , I. Stirbu , A.‐J. R. Roskam , et al. 2008. “Socioeconomic Inequalities in Health in 22 European Countries.” New England Journal of Medicine 358: 2468–2481. 10.1056/nejmsa0707519. 18525043

[ejp70441-bib-0016] Maraschini, A. , M. Tenti , W. Raffaeli , et al. 2025. “Chronic Pain Prevalence and Psychosocial Burden in the Italian Population From the 2019 European Health Interview Survey.” International Journal of Environmental Research and Public Health 22: 1395. 10.3390/ijerph22091395. PMC12469650 41007538

[ejp70441-bib-0017] Moreno‐Betancur, M. , A. Latouche , G. Menvielle , A. E. Kunst , and G. Rey . 2015. “Relative Index of Inequality and Slope Index of Inequality: A Structured Regression Framework for Estimation.” Epidemiology 26: 518–527. 10.1097/EDE.0000000000000311. 26000548

[ejp70441-bib-0018] Neogi, T. 2013. “The Epidemiology and Impact of Pain in Osteoarthritis.” Osteoarthritis and Cartilage 21: 1145–1153. 10.1016/j.joca.2013.03.018. PMC3753584 23973124

[ejp70441-bib-0019] Pearce, N. 2004. “Effect Measures in Prevalence Studies.” Environmental Health Perspectives 112: 1047–1050. 10.1289/ehp.6927. PMC1247374 15238274

[ejp70441-bib-0020] Pereira Silva, A. M. , L. A. Gimeno‐Feliu , and J. A. Lopez‐Rodriguez . 2025. “The Burden of Inequity: Income Related Disparities in Cardiovascular Risk Factors in Europe.” Public Health 248: 105978. 10.1016/j.puhe.2025.105978. 41076957

[ejp70441-bib-0021] Raja, S. N. , D. B. Carr , M. Cohen , et al. 2020. “The Revised International Association for the Study of Pain Definition of Pain: Concepts, Challenges, and Compromises.” Pain 161: 1976–1982. 10.1097/j.pain.0000000000001939. PMC7680716 32694387

[ejp70441-bib-0022] Regidor, E. 2004. “Measures of Health Inequalities: Part 2.” Journal of Epidemiology and Community Health 58: 900–903. 10.1136/jech.2004.023036. PMC1732621 15483304

[ejp70441-bib-0023] Solé, E. , M. Racine , C. Tomé‐Pires , S. Galán , M. P. Jensen , and J. Miró . 2020. “Social Factors, Disability, and Depressive Symptoms in Adults With Chronic Pain.” Clinical Journal of Pain 36: 371–378. 10.1097/AJP.0000000000000815. 32040011

[ejp70441-bib-0024] Steinmetz, J. D. , G. T. Culbreth , L. M. Haile , et al. 2023. “Global, Regional, and National Burden of Osteoarthritis, 1990–2020 and Projections to 2050: A Systematic Analysis for the Global Burden of Disease Study 2021.” Lancet Rheumatology 5: e508–e522. 10.1016/S2665-9913(23)00163-7. PMC10477960 37675071

[ejp70441-bib-0025] Thompson, M. L. , J. E. Myers , and D. Kriebel . 1998. “Prevalence Odds Ratio or Prevalence Ratio in the Analysis of Cross Sectional Data: What is to be Done?” Occupational and Environmental Medicine 55: 272–277. 10.1136/oem.55.4.272. PMC1757577 9624282

[ejp70441-bib-0026] Todd, A. , C. L. McNamara , M. Balaj , et al. 2019. “The European Epidemic: Pain Prevalence and Socioeconomic Inequalities in Pain Across 19 European Countries.” European Journal of Pain 23: 1425–1436. 10.1002/ejp.1409. 31038816

[ejp70441-bib-0027] Van Schoor, N. M. , S. Zambon , M. V. Castell , et al. 2016. “Impact of Clinical Osteoarthritis of the Hip, Knee and Hand on Self‐Rated Health in Six European Countries: The European Project on OSteoArthritis.” Quality of Life Research 25: 1423–1432. 10.1007/s11136-015-1171-8. PMC4870285 26547441

[ejp70441-bib-0028] Von Elm, E. , D. G. Altman , M. Egger , S. J. Pocock , P. C. Gøtzsche , and J. P. Vandenbroucke . 2008. “The Strengthening the Reporting of Observational Studies in Epidemiology (STROBE) Statement: Guidelines for Reporting Observational Studies.” Journal of Clinical Epidemiology 61: 344–349. 10.1016/j.jclinepi.2007.11.008. 18313558

[ejp70441-bib-0029] WHO . 2023. Osteoarthritis. World Health Organization.

[ejp70441-bib-0030] Yan, H. , J. Guo , W. Zhou , C. Dong , and J. Liu . 2022. “Health‐Related Quality of Life in Osteoarthritis Patients: A Systematic Review and Meta‐Analysis.” Psychology, Health & Medicine 27: 1859–1874. 10.1080/13548506.2021.1971725. 34465255

[ejp70441-bib-0031] Yeamans, S. , Á. Gil‐De‐Miguel , V. Hernández‐Barrera , and P. Carrasco‐Garrido . 2026. “Self‐Medication and Pain in the European Union: Gender Differences and Associated Factors.” Preventive Medical Reports 66: 103488. 10.1016/j.pmedr.2026.103488. PMC13156551 42109499

[ejp70441-bib-0032] Zimmer, Z. , K. Fraser , H. Grol‐Prokopczyk , and A. Zajacova . 2022. “A Global Study of Pain Prevalence Across 52 Countries: Examining the Role of Country‐Level Contextual Factors.” Pain 163: 1740–1750. 10.1097/j.pain.0000000000002557. PMC9198107 35027516

[ejp70441-bib-0033] Zimmer, Z. , A. Zajacova , K. Fraser , D. Powers , and H. Grol‐Prokopczyk . 2023. “A Global Comparative Study of Wealth‐Pain Gradients: Investigating Individual‐ and Country‐Level Associations.” Dialogues Health 2: 100122. 10.1016/j.dialog.2023.100122. PMC10718570 38099153

[ejp70441-bib-0034] Zou, G. 2004. “A Modified Poisson Regression Approach to Prospective Studies With Binary Data.” American Journal of Epidemiology 159: 702–706. 10.1093/aje/kwh090. 15033648

