# Supplementary material for: The Social Gradient of Pain in Europe: Income‐Related Inequities and the Modifying Role of Osteoarthritis
Source: Eur J Pain. 2026 Nov 1;30(10):e70441. doi: 10.1002/ejp.70441 (PMC13724799; doi:10.1002/ejp.70441)
Supplement: Supplementary file 1 [file EJP-30-0-s001.docx]

## **Supplementary material**

# **Table S1. Distribution of the analytical sample by country and presence of pain in the past 4 weeks**

| **Country** | **N** | **n without pain** | **No pain, weighted %** | **n with pain** | **Pain, weighted %** |
| --- | --- | --- | --- | --- | --- |
| AT | 14,980 | 6,435 | 43.5 | 8,545 | 56.5 |
| BE | 6,633 | 2,575 | 39.0 | 4,058 | 61.0 |
| BG | 6,732 | 3,495 | 51.3 | 3,237 | 48.7 |
| CY | 5,523 | 3,356 | 55.0 | 2,167 | 45.0 |
| CZ | 7,453 | 3,377 | 41.6 | 4,076 | 58.4 |
| DE | 21,930 | 9,120 | 41.7 | 12,810 | 58.3 |
| DK | 4,917 | 1,614 | 32.3 | 3,303 | 67.7 |
| EE | 4,659 | 1,753 | 38.3 | 2,906 | 61.7 |
| EL | 7,591 | 4,360 | 53.5 | 3,231 | 46.5 |
| ES | 19,666 | 11,202 | 56.0 | 8,464 | 44.0 |
| FI | 4,863 | 1,406 | 28.7 | 3,457 | 71.3 |
| FR | 13,559 | 5,250 | 38.6 | 8,309 | 61.4 |
| HR | 3,103 | 1,086 | 35.6 | 2,017 | 64.4 |
| HU | 5,301 | 2,713 | 52.5 | 2,588 | 47.5 |
| IE | 1,977 | 987 | 52.1 | 990 | 47.9 |
| IS | 3,478 | 1,050 | 30.6 | 2,428 | 69.4 |
| IT | 42,284 | 24,348 | 57.5 | 17,936 | 42.5 |
| LT | 4,103 | 1,474 | 39.2 | 2,629 | 60.8 |
| LU | 3,020 | 967 | 29.1 | 2,053 | 70.9 |
| LV | 5,574 | 2,840 | 51.2 | 2,734 | 48.8 |
| MT | 3,794 | 2,127 | 52.0 | 1,667 | 48.0 |
| NL | 7,569 | 2,867 | 37.8 | 4,702 | 62.2 |
| NO | 7,332 | 4,128 | 55.9 | 3,204 | 44.1 |
| PL | 12,253 | 4,837 | 37.5 | 7,416 | 62.5 |
| PT | 13,737 | 5,126 | 36.4 | 8,611 | 63.6 |
| RO | 14,805 | 6,213 | 38.8 | 8,592 | 61.2 |
| RS | 8,247 | 5,163 | 62.2 | 3,084 | 37.8 |
| SE | 8,697 | 2,170 | 26.5 | 6,527 | 73.5 |
| SI | 9,126 | 3,080 | 33.5 | 6,046 | 66.5 |
| SK | 5,344 | 2,544 | 45.7 | 2,800 | 54.3 |

**Note.** N and n refer to unweighted crude frequencies. Percentages are weighted estimates using wt_rescaled_m1. The multivariable analytical sample included 278,250 participants from 30 European countries. AT: Austria; BE: Belgium; BG: Bulgaria; CY: Cyprus; CZ: Czechia; DE: Germany; DK: Denmark; EE: Estonia; EL: Greece; ES: Spain; FI: Finland; FR: France; HR: Croatia; HU: Hungary; IE: Ireland; IS: Iceland; IT: Italy; LT: Lithuania; LU: Luxembourg; LV: Latvia; MT: Malta; NL: Netherlands; NO: Norway; PL: Poland; PT: Portugal; RO: Romania; RS: Serbia; SE: Sweden; SI: Slovenia; SK: Slovakia.

# **Table S2. Sensitivity analysis using logistic regression of the association between household income quintile, osteoarthritis, and pain in the past 4 weeks**

| **Variable** | **aOR** | **95% CI** | **p-value** |
| --- | --- | --- | --- |
| **Household income quintile** |  |  |  |
| Q1, lowest | 1.000 | Reference | — |
| Q2 | 0.967 | 0.932–1.003 | 0.069 |
| Q3 | 0.922 | 0.890–0.956 | <0.001 |
| Q4 | 0.890 | 0.858–0.923 | <0.001 |
| Q5, highest | 0.840 | 0.809–0.872 | <0.001 |
| **Osteoarthritis** |  |  |  |
| Without osteoarthritis | 1.000 | Reference | — |
| With osteoarthritis | 4.812 | 4.420–5.240 | <0.001 |
| **Interaction between household income quintile and osteoarthritis** |  |  |  |
| Q1, lowest × without osteoarthritis | 1.000 | Reference | — |
| Q1, lowest × with osteoarthritis | 1.000 | Reference | — |
| Q2 × without osteoarthritis | 1.000 | Reference | — |
| Q2 × with osteoarthritis | 0.960 | 0.858–1.074 | 0.473 |
| Q3 × without osteoarthritis | 1.000 | Reference | — |
| Q3 × with osteoarthritis | 0.941 | 0.840–1.054 | 0.290 |
| Q4 × without osteoarthritis | 1.000 | Reference | — |
| Q4 × with osteoarthritis | 0.851 | 0.760–0.954 | 0.006 |
| Q5, highest × without osteoarthritis | 1.000 | Reference | — |
| Q5, highest × with osteoarthritis | 0.886 | 0.789–0.994 | 0.040 |
| **Educational attainment** |  |  |  |
| Low: ISCED 0–2 | 1.113 | 1.082–1.145 | <0.001 |
| Medium: ISCED 3–4 | 1.000 | Reference | — |
| High: ISCED 5–8 | 0.935 | 0.910–0.960 | <0.001 |
| **Employment status** |  |  |  |
| Employed | 1.110 | 1.058–1.165 | <0.001 |
| Unemployed | 1.000 | Reference | — |
| Retired | 1.289 | 1.216–1.367 | <0.001 |
| Unable to work | 3.152 | 2.825–3.515 | <0.001 |
| Student | 1.029 | 0.958–1.105 | 0.433 |
| Domestic tasks | 0.959 | 0.900–1.023 | 0.202 |
| Other | 1.163 | 1.067–1.267 | 0.001 |
| **Body mass index** |  |  |  |
| Underweight | 1.065 | 0.989–1.148 | 0.095 |
| Normal weight | 1.000 | Reference | — |
| Overweight | 1.185 | 1.157–1.213 | <0.001 |
| Obesity class I | 1.436 | 1.388–1.486 | <0.001 |
| Obesity class II | 1.613 | 1.512–1.721 | <0.001 |
| Obesity class III | 1.851 | 1.652–2.074 | <0.001 |
| **Current smoking** |  |  |  |
| Non-smoker | 1.000 | Reference | — |
| Current smoker | 1.068 | 1.042–1.096 | <0.001 |
| **Depression in the past 12 months** |  |  |  |
| No depression | 1.000 | Reference | — |
| Depression in the past 12 months | 3.017 | 2.864–3.178 | <0.001 |
| **Sex** |  |  |  |
| Men | 1.000 | Reference | — |
| Women | 1.438 | 1.407–1.470 | <0.001 |
| **Age group** |  |  |  |
| 18–29 years | 1.000 | Reference | — |
| 30–44 years | 1.265 | 1.214–1.317 | <0.001 |
| 45–59 years | 1.661 | 1.596–1.730 | <0.001 |
| 60–74 years | 1.748 | 1.665–1.836 | <0.001 |
| ≥75 years | 2.377 | 2.239–2.523 | <0.001 |
| **Model statistics** |  |  |  |
| **N** | **278,250** |  |  |
| **Wald χ²** | **23,932.575** |  |  |
| **Overall p-value** | **<0.001** |  |  |
| **Pseudo R²** | **0.135** |  |  |

**Note.** aOR: adjusted odds ratio; 95% CI: 95% confidence interval. Logistic regression was used as a sensitivity analysis. The model included the interaction between household income quintile and osteoarthritis status, adjusted for educational attainment, employment status, body mass index, current smoking, depression in the past 12 months, sex, and age, and included country fixed effects, which are not shown for readability. Q1 and Q5 correspond to the lowest and highest household income quintiles, respectively. Coefficients were exponentiated and are presented as aORs. The analytical sample included 278,250 participants with complete data for all model variables.

# **Table S3. Relative inequality in pain by household income quintile and country: adjusted RII from the final multivariable model**

| **Country** | **N** | **RII** | **95% CI** | **p-value** |
| --- | --- | --- | --- | --- |
| AT | 14.980 | 1.139 | 1.063–1.221 | <0.001 |
| BE | 6.633 | 0.944 | 0.838–1.063 | 0.342 |
| BG | 6.732 | 0.995 | 0.887–1.116 | 0.934 |
| CY | 5.523 | 1.013 | 0.872–1.177 | 0.864 |
| CZ | 7,453 | 1.180 | 1.056–1.319 | 0.004 |
| DE | 21,930 | 1.094 | 1.036–1.156 | 0.001 |
| DK | 4,917 | 0.961 | 0.887–1.041 | 0.334 |
| EE | 4,659 | 1.023 | 0.917–1.141 | 0.690 |
| EL | 7,591 | 1.038 | 0.883–1.219 | 0.651 |
| ES | 19,666 | 0.991 | 0.920–1.067 | 0.807 |
| FI | 4,863 | 1.071 | 0.989–1.159 | 0.093 |
| FR | 13,559 | 1.107 | 1.044–1.173 | <0.001 |
| HR | 3,103 | 0.969 | 0.860–1.092 | 0.607 |
| HU | 5,301 | 1.105 | 0.970–1.259 | 0.134 |
| IE | 1,977 | 0.927 | 0.714–1.203 | 0.570 |
| IS | 3,478 | 1.009 | 0.895–1.139 | 0.879 |
| IT | 42,284 | 1.010 | 0.958–1.065 | 0.700 |
| LT | 4,103 | 1.110 | 0.977–1.262 | 0.109 |
| LU | 3,020 | 0.907 | 0.796–1.034 | 0.145 |
| LV | 5,574 | 1.029 | 0.896–1.182 | 0.687 |
| MT | 3,794 | 1.043 | 0.876–1.243 | 0.634 |
| NL | 7,569 | 1.058 | 0.977–1.145 | 0.165 |
| NO | 7,332 | 1.193 | 1.062–1.341 | 0.003 |
| PL | 12,253 | 1.101 | 1.029–1.177 | 0.005 |
| PT | 13,737 | 1.032 | 0.952–1.119 | 0.446 |
| RO | 14,805 | 1.141 | 1.082–1.204 | <0.001 |
| RS | 8,247 | 0.868 | 0.768–0.980 | 0.023 |
| SE | 8,697 | 1.114 | 1.049–1.184 | <0.001 |
| SI | 9,126 | 1.091 | 1.012–1.175 | 0.023 |
| SK | 5,344 | 1.165 | 1.044–1.301 | 0.006 |

**Note.** The RII expresses the adjusted prevalence ratio comparing the lowest-income end with the highest-income end within each country, estimated using the household income-quintile ridit score. Models were adjusted for osteoarthritis, educational attainment, employment status, body mass index, current smoking, depression in the past 12 months, sex, and age. RII values >1 show a higher adjusted prevalence of pain at the lowest-income end; RII values <1 show a reversed gradient. Q1 corresponds to the lowest household income quintile and Q5 to the highest household income quintile. N refers to unweighted frequencies.

# **Table S4. Absolute inequality in pain by household income quintile and country: adjusted SII from the final multivariable model**

| **Country** | **N** | **Adjusted prevalence Q1** | **Adjusted prevalence Q5** | **SII, pp** | **95% CI** | **p-value** |
| --- | --- | --- | --- | --- | --- | --- |
| AT | 14,980 | 0.592 | 0.533 | 5.86 | 2.77–8.94 | <0.001 |
| BE | 6,633 | 0.595 | 0.623 | −2.81 | −8.58–2.96 | 0.341 |
| BG | 6,732 | 0.486 | 0.488 | −0.19 | −4.65–4.28 | 0.934 |
| CY | 5,523 | 0.452 | 0.447 | 0.47 | −4.90–5.84 | 0.864 |
| CZ | 7,453 | 0.613 | 0.537 | 7.61 | 2.58–12.63 | 0.003 |
| DE | 21,930 | 0.609 | 0.566 | 4.23 | 1.64–6.82 | 0.001 |
| DK | 4,917 | 0.666 | 0.687 | −2.13 | −6.46–2.19 | 0.334 |
| EE | 4,659 | 0.623 | 0.612 | 1.10 | −4.30–6.50 | 0.690 |
| EL | 7,591 | 0.472 | 0.458 | 1.38 | −4.61–7.38 | 0.651 |
| ES | 19,666 | 0.439 | 0.442 | −0.33 | −2.95–2.29 | 0.807 |
| FI | 4,863 | 0.733 | 0.694 | 3.89 | −0.64 8.43 | 0.093 |
| FR | 13,559 | 0.638 | 0.588 | 4.98 | 2.13–7.82 | <0.001 |
| HR | 3,103 | 0.636 | 0.652 | −1.62 | −7.78–4.55 | 0.607 |
| HU | 5,301 | 0.493 | 0.455 | 3.79 | −1.16–8.74 | 0.133 |
| IE | 1,977 | 0.467 | 0.496 | −2.91 | −13.01–7.19 | 0.572 |
| IS | 3,478 | 0.697 | 0.692 | 0.52 | −6.17–7.22 | 0.879 |
| IT | 42,284 | 0.426 | 0.423 | 0.35 | −1.44–2.15 | 0.700 |
| LT | 4,103 | 0.631 | 0.580 | 5.07 | −1.10–11.20 | 0.107 |
| LU | 3,020 | 0.685 | 0.740 | −5.54 | −13.00–1.90 | 0.146 |
| LV | 5,574 | 0.492 | 0.481 | 1.11 | −4.28–6.50 | 0.687 |
| MT | 3,794 | 0.487 | 0.471 | 1.62 | −5.05–8.30 | 0.633 |
| NL | 7,569 | 0.637 | 0.609 | 2.80 | −1.16–6.77 | 0.166 |
| NO | 7,332 | 0.473 | 0.411 | 6.24 | 2.12–10.36 | 0.003 |
| PL | 12,253 | 0.645 | 0.597 | 4.77 | 1.45–8.09 | 0.005 |
| PT | 13,737 | 0.643 | 0.627 | 1.60 | −2.51–5.71 | 0.446 |
| RO | 14,805 | 0.642 | 0.578 | 6.43 | 3.84–9.02 | <0.001 |
| RS | 8,247 | 0.356 | 0.399 | −4.27 | −7.95–−0.55 | 0.023 |
| SE | 8,697 | 0.775 | 0.711 | 6.43 | 2.80–10.06 | <0.001 |
| SI | 9,126 | 0.690 | 0.644 | 4.63 | 0.64–8.62 | 0.023 |
| SK | 5,344 | 0.576 | 0.510 | 6.64 | 1.90–11.38 | 0.006 |

**Note.** pp= percentage points. The SII expresses the adjusted absolute difference in pain prevalence between the lowest-income end and the highest-income end within each country, in percentage points, estimated using the household income-quintile ridit score. Positive values show a higher adjusted prevalence of pain at the lowest-income end; negative values show a reversed gradient. Q1 corresponds to the lowest household income quintile and Q5 to the highest household income quintile. Models were adjusted for osteoarthritis, educational attainment, employment status, body mass index, current smoking, depression in the past 12 months, sex, and age. N refers to unweighted frequencies.

# **Table S5. Relative inequality in pain by household income quintile, country, and osteoarthritis status: adjusted RII from the final multivariable model**

| **Country** | **N without OA** | **RII without OA** | **95% CI** | **p-value** | **N with OA** | **RII with OA** | **95% CI** | **p-value** |
| --- | --- | --- | --- | --- | --- | --- | --- | --- |
| AT | 12,602 | 1.133 | 1.042–1.230 | 0.003 | 2,378 | 1.104 | 1.019–1.190 | 0.016 |
| BE | 5,273 | 0.960 | 0.826–1.110 | 0.590 | 1,360 | 0.971 | 0.841–1.120 | 0.685 |
| BG | 6,225 | 0.964 | 0.847–1.090 | 0.576 | 507 | 1.135 | 0.966–1.330 | 0.124 |
| CY | 4,803 | 1.051 | 0.865–1.270 | 0.618 | 720 | 0.947 | 0.805–1.110 | 0.514 |
| CZ | 6,061 | 1.168 | 1.013–1.340 | 0.033 | 1,392 | 1.105 | 0.980–1.240 | 0.103 |
| DE | 17,690 | 1.072 | 0.999–1.150 | 0.054 | 4,240 | 1.078 | 1.017–1.140 | 0.012 |
| DK | 3,817 | 0.940 | 0.844–1.040 | 0.255 | 1,100 | 0.974 | 0.907–1.040 | 0.467 |
| EE | 4,351 | 0.997 | 0.888–1.110 | 0.958 | 308 | 1.172 | 0.961–1.430 | 0.118 |
| EL | 7,076 | 1.049 | 0.875–1.250 | 0.604 | 515 | 1.129 | 0.966–1.310 | 0.128 |
| ES | 15,849 | 0.970 | 0.876–1.070 | 0.556 | 3,817 | 1.057 | 0.972–1.140 | 0.193 |
| FI | 3,652 | 1.130 | 1.022–1.240 | 0.017 | 1,211 | 0.982 | 0.887–1.080 | 0.725 |
| FR | 10,176 | 1.142 | 1.053–1.230 | 0.001 | 3,383 | 1.014 | 0.959–1.070 | 0.624 |
| HR | 2,660 | 0.954 | 0.825–1.100 | 0.526 | 443 | 1.070 | 0.952–1.200 | 0.258 |
| HU | 4,190 | 1.065 | 0.901–1.250 | 0.462 | 1,111 | 1.080 | 0.932–1.250 | 0.307 |
| IE | 1,932 | 0.919 | 0.704–1.200 | 0.537 | 45 | 0.753 | 0.534–1.060 | 0.106 |
| IS | 2,814 | 0.990 | 0.855–1.140 | 0.894 | 664 | 1.052 | 0.914–1.210 | 0.481 |
| IT | 33,327 | 0.938 | 0.870–1.010 | 0.097 | 8,957 | 1.062 | 1.003–1.120 | 0.039 |
| LT | 3,625 | 1.103 | 0.953–1.270 | 0.188 | 478 | 1.042 | 0.927–1.170 | 0.494 |
| LU | 2,498 | 0.838 | 0.711–0.980 | 0.036 | 522 | 1.093 | 0.975–1.220 | 0.127 |
| LV | 4,886 | 0.986 | 0.840–1.150 | 0.864 | 688 | 1.058 | 0.874–1.280 | 0.565 |
| MT | 3,527 | 1.110 | 0.912–1.350 | 0.299 | 267 | — | — | — |
| NL | 6,216 | 1.063 | 0.965–1.170 | 0.214 | 1,353 | 0.972 | 0.888–1.060 | 0.538 |
| NO | 6,284 | 1.181 | 1.030–1.350 | 0.017 | 1,048 | 1.201 | 1.015–1.420 | 0.033 |
| PL | 9,484 | 1.105 | 1.006–1.210 | 0.037 | 2,769 | 1.004 | 0.951–1.060 | 0.875 |
| PT | 9,477 | 0.964 | 0.857–1.080 | 0.538 | 4,260 | 1.073 | 0.995–1.150 | 0.068 |
| RO | 14,070 | 1.153 | 1.088–1.220 | <0.001 | 735 | 1.000 | 0.997–1.000 | 0.979 |
| RS | 7,646 | 0.814 | 0.709–0.930 | 0.003 | 601 | 1.093 | 0.916–1.300 | 0.325 |
| SE | 7,282 | 1.146 | 1.069–1.220 | <0.001 | 1,415 | 0.978 | 0.922–1.030 | 0.462 |
| SI | 8,641 | 1.097 | 1.012–1.180 | 0.024 | 485 | 1.044 | 0.922–1.180 | 0.499 |
| SK | 4,393 | 1.170 | 1.011–1.350 | 0.035 | 951 | 1.088 | 0.988–1.190 | 0.086 |

**Note.** OA= osteoarthritis**.** The RII estimates the adjusted prevalence ratio between the lowest- and highest-income ends within each country and osteoarthritis stratum, using the household income-quintile ridit score. RII values >1 show higher adjusted pain prevalence at the lowest-income end; values <1 show a reversed gradient. Models were adjusted for educational attainment, employment status, body mass index, current smoking, depression in the past 12 months, sex, and age. N refers to unweighted frequencies. In Malta, the RII in the osteoarthritis stratum was not estimable.

# **Figure S1a. Relative inequality in bodily pain among participants with osteoarthritis**

**Note:** Country-specific adjusted Relative Index of Inequality (RII) for bodily pain among participants with osteoarthritis. The color scale ranges from 0.753 to 1.201, with 1.000 indicating no socioeconomic gradient. Values above 1 indicate higher pain prevalence among lower-income groups, whereas values below 1 indicate an inverse socioeconomic gradient in pain. Malta had no valid country-specific estimate for this stratum.

# **Figure S1b. Relative inequality in bodily pain across European countries**

**Note.** Country-specific adjusted Relative Index of Inequality (RII) for bodily pain from the final multivariable model. The color scale ranges from 0.868 to 1.193, with 1.000 indicating no socioeconomic gradient. Values above 1 indicate higher pain prevalence among lower-income groups, whereas values below 1 indicate an inverse socioeconomic gradient in pain.

# **Figure S1c. Relative inequality in bodily pain among participants without osteoarthritis**

**Note**: Country-specific adjusted Relative Index of Inequality (RII) for bodily pain among participants without osteoarthritis. Values above 1 indicate higher pain prevalence among lower-income groups, whereas values below 1 indicate an inverse socioeconomic gradient in pain.

# **Figure S2. Forest plot of the Relative Index of Inequality for pain by country**

**Note.** Points indicate country-specific adjusted RII estimates, with horizontal bars showing 95% CIs. The dashed vertical line represents no relative inequality (RII = 1). RII > 1 indicates higher pain prevalence at the lowest-income end; RII < 1 indicates an inverse gradient. Models were adjusted for osteoarthritis, education, employment status, body mass index, current smoking, depression, sex, and age.

# **Figure S3. Forest plot of the Slope Index of Inequality for pain by country**

**Note.** Points indicate country-specific adjusted SII estimates, with horizontal bars showing 95% CIs. The dashed vertical line represents no absolute inequality (SII = 0). Positive values indicate higher pain prevalence at the lowest-income end, whereas negative values indicate an inverse gradient. Estimates were adjusted for osteoarthritis, education, employment status, body mass index, current smoking, depression, sex, and age.

# **Figure S4. Forest plot of the Relative Index of Inequality for pain by country, stratified by osteoarthritis status**

**Note.** Points represent country-specific adjusted RII estimates, and horizontal bars indicate 95% confidence intervals, stratified by osteoarthritis status. The dashed vertical line represents no relative inequality (RII = 1). RII values > 1 indicate a higher adjusted prevalence of pain at the lowest-income end of the distribution, whereas RII values < 1 indicate an inverse gradient. Models were adjusted for education, employment status, body mass index, current smoking, depression in the past 12 months, sex, and age. In Malta, the RII for participants with osteoarthritis was not estimable.
